# Messaging Mask Wearing During the COVID-19 Crisis: Ideological Differences

**DOI:** 10.1017/XPS.2020.15

**Published:** 2020-05-27

**Authors:** Stephen M. Utych

**Affiliations:** Boise State University, Boise, Idaho, USA

**Keywords:** COVID-19, ideology, regulatory focus theory

## Abstract

As the U.S. Government works to slow the spread of the novel coronavirus, messaging is important in getting individuals to comply with public health recommendations, especially as the response from the public seems to be polarized along partisan and ideological lines. Using a recent Centers for Disease Control recommendation of wearing facemasks, I use Regulatory Focus Theory to predict that conservatives will be more responsive to messages related to promotion, while liberals are more responsive to messages related to prevention. Using a pre-registered experimental design, I find no evidence that prevention messages influence attitudes toward mask wearing. Promotion messages, however, cause conservatives to become less supportive of mask wearing, in contrast to theoretical predictions. These findings suggest that, related to messaging about mask wearing, strong ideological differences do not emerge related to the focus of the message.

As the COVID-19 epidemic has spread around the world, governments are challenged with producing messages that encourage citizens to partake in behaviors designed to slow the spread of the disease, which will help to mitigate the negative impacts of the disease. In the USA, this has proven especially challenging, with different states offering drastically different policy solutions to encourage compliance with recommendations of health officials, ranging from very strong “shelter in place” orders, to recommendations with no enforcement mechanism.^1^ Additionally, the federal government is producing additional information, mostly derived from daily press briefings on the novel coronavirus. On Friday, April 3, the Centers for Disease Control (CDC) issued an official recommendation for Americans to wear cloth masks when leaving their home.^2^ While not a requirement, the CDC strongly recommends the use of these masks and has provided a guide for citizens to create them.

While masks are effective at slowing the spread of novel viruses, like the H5N1 avian flu, citizens in areas where mask wearing is not common are generally not very compliant with these recommendations (MacIntyre et al., 2009). How, then, can the USA encourage individuals to adopt this practice? I expect that if the government is able to carefully tailor its messages to individuals about the potential benefits of mask wearing, compliance can be increased. Strong and effective messaging from the government can minimize the impacts of crises, like natural disasters (Coombs, 1995). Importantly, effective messaging can increase compliance with government recommendations (McAdams and Nadler, 2005). Related to the COVID-19 pandemic, government messaging can play an important role. In Italy, compliance with government messages is generally high, even among individuals who are not inclined to trust the government (Barari et al., 2020). In the USA, compliance is also high (Pew Research Center, 2020), though differences emerge based on how the government messages the threat. When risks to older Americans are highlighted, younger Americans are marginally *less* complaint with government recommendations, but messages about younger Americans help individuals see COVID-19 as a greater threat (Utych and Fowler, 2020). While compliance with recommendations is high, it is clear that messaging strategies from the government can influence these levels of compliance.

I examine how government messages interact with individual ideology to encourage the use of cloth masks, per recent CDC recommendations. Americans are deeply polarized along partisan and ideological lines (Mason, 2018), and this effect extends to response to the COVID-19 crisis. Preliminary data find that conservatives and Republicans are less likely to report intention to conduct simple activities to prevent the spread of the disease, like washing their hands and covering their cough, than liberals and Democrats (Fowler and Utych, 2020). I argue that the types of messages individuals receive can help bridge this partisan and ideological divide.

Regulatory Focus Theory (RFT) argues that individual motivation is driven by two concepts – promotion and prevention (Higgins, 1997). Promotion focuses on achieving positive outcomes, while prevention focuses on avoiding negative outcomes (Higgins, 1997). Importantly, these goals are connected to political ideology – as conservatives tend to achieve happiness through prevention, while liberals achieve happiness through promotion (Choma, Busseri and Sadava, 2009). Liberals tend to show a sensitivity to detecting positive affect, while conservatives show a sensitivity to detecting negative affect (Tomkins, 1965). When information is framed as a gain, it can be seen as a promotional focus, while information framed as a loss, it is seen as a prevention focus (Lee and Aaker, 2004). This suggests that *how* wearing masks in public is framed by political elites could increase compliance, but that these frames are likely to work differently on liberals and conservatives.

RFT provides two clear hypotheses with respect to government messaging on wearing surgical masks. The first hypothesis is that *messages related to promotion will increase the likelihood that liberals comply with wearing facemasks.* The second hypothesis predicts that *messages related to prevention will increase the likelihood that conservatives comply with wearing facemasks.*

## Experimental Design

Participants were recruited from Amazon’s Mechanical Turk (MTurk) on April 15, 2020.^3^ Considering worries about bots on MTurk, or automated, non-serious responses designed solely to collect payment, care was taken per Moss and Litman (2018) to screen out bots or non-US respondents.^4^ Given that general compliance with regulations is high, it is likely that the effects of messaging will be small. Using a Cohen’s *d* of a small effect, this would require 139 participants per experimental condition, of which there are 3. However, given that only about 1/3 of respondents on MTurk identify as conservative, I recruited 3 times that may participants to ensure enough conservatives per group. This gives a total of 1,251 respondents recruited to participate in this study. I conducted a post data collection, pre-analysis screening for bad IP addresses per Kennedy et al. (2018), which left a total of 1,216 respondents.

The subject pool was typical of MTurk samples. Age of participants ranged from 18 to 79, with a mean of 38.6. 48% of the sample identified as female and 76% as white. The sample was highly educated, as 60.2% had a bachelor’s degree or higher, and also skewed liberal, with 54.3% identifying as liberal, 17.2% as moderate, and 28.5% as conservative. After answering a brief series of demographics questions, participants were randomly assigned to one of three experimental conditions – a *control* condition, a *prevention* condition, and a *promotion* condition.

In the control condition, individuals were presented with purely informational text about the CDC’s mask recommendation. In the prevention condition, they were presented information that this recommendation will help prevent negative health and economic outcomes, while in the promotion condition, they were presented information that this recommendation will lead to positive health and economic outcomes. The text of these treatments is below.

Control:

*The CDC has recently recommended that all Americans wear a facial mask when leaving their homes, in order to prevent the spread of the coronavirus.*

Prevention:

*The CDC has recently recommended that all Americans wear a facial mask when leaving their homes, in order to prevent the spread of the coronavirus.*

*Following this recommendation will help us prevent Americans from dying and prevent our economy from entering a long-term recession.*

Promotion:

*The CDC has recently recommended that all Americans wear a facial mask when leaving their homes, in order to prevent the spread of the coronavirus.*

*Following this recommendation will help us to keep Americans healthy and allow our economy to return to normal more quickly.*

After reading this text, individuals responded to four dependent measures, measured on a seven-point scale, ranging from strongly disagree (1) to strongly agree (7). The first is a behavioral intention question – “I will wear a mask every time I leave my home.” The next two are attitudinal questions – “It is best for society if everyone wears a mask when they leave their home” and “I don’t think wearing a mask will impact the spread of the coronavirus.” A final question, asking participants whether they would like to get more information about purchasing face masks, was asked as a yes or no question, to better get at behavior, rather than behavioral intention.

## Analysis

These analyses follow a pre-analysis plan, registered with Open Science Framework (OSF) at https://osf.io/gbpnq/. Recall that both hypotheses are conditional on ideology – promotion messages should work better with liberals, and prevention messages better with conservatives. Three sets of analyses were pre-registered in this plan.

Since RFT predictions about ideology focus primarily on *direction* of ideology rather than strength (Choma, Busseri and Sadava, 2009), I first separate ideology between liberals and conservatives. Ideology was asked on the standard American National Election Study (ANES) seven-point scale, allowing respondents to select responses ranging from “Very conservative” to “Very liberal,” with an option for respondents to select that they “haven’t thought much about this.”

Those who report their ideology as “moderate/middle of the road” or respond that they “haven’t thought much about this” are excluded from analysis. Respondents who answer “Very conservative,” “conservative,” or “somewhat conservative” are coded as “conservative,” while those who answer “very liberal,” “liberal,” or “somewhat liberal” will be coded as “liberal.” This creates a dichotomous measure of ideology, with individuals coded as either liberal (1) or conservative (0). This measure is then interacted with each of the treatments, with the control group serving as reference. Of course, RFT provides relatively unclear predictions about how *strength* of ideology might impact the power of these types of messages on attitudes and behaviors. As an additional set of analyses, I interact the full ideological measure (ranging from very conservative to very liberal) with the treatments, rather than the dichotomous measure. This allows for the inclusion of ideological moderates in these analyses but still requires excluding those who do not place themselves on the ideological scale.

As a last set of analyses, I examine these relationships conditional on a match between a participant’s stated partisanship and the partisanship of their governor, given that recent work finds that individuals are less likely to comply with measures when they do not share partisanship with their governor (Cornelson and Miloucheva, 2020). While there is little theory available to suggest that types of messages might interact with this, it is an important exploratory analysis given the findings of Cornelson and Miloucheva (2020), which are directly related to COVID-19. This will create a three-way interaction between the treatments, respondent ideology, and respondent co-partisanship with their state’s governor. Individual partisanship is based off of the standard two-pronged ANES partisanship question, including partisan leaners. This is matched with an individual’s self-reported state of residence and the partisanship of the current governor of that state.

Given that this research design relies upon an observed moderator of ideology, I include controls for observed covariates (Kam and Trussler, 2017) – age, gender, race, education, and whether a respondent has a co-partisan governor or not, since research shows this affects compliance with COVID-19 related regulations (Cornelson and Miloucheva, 2020).

**Figure 1:**
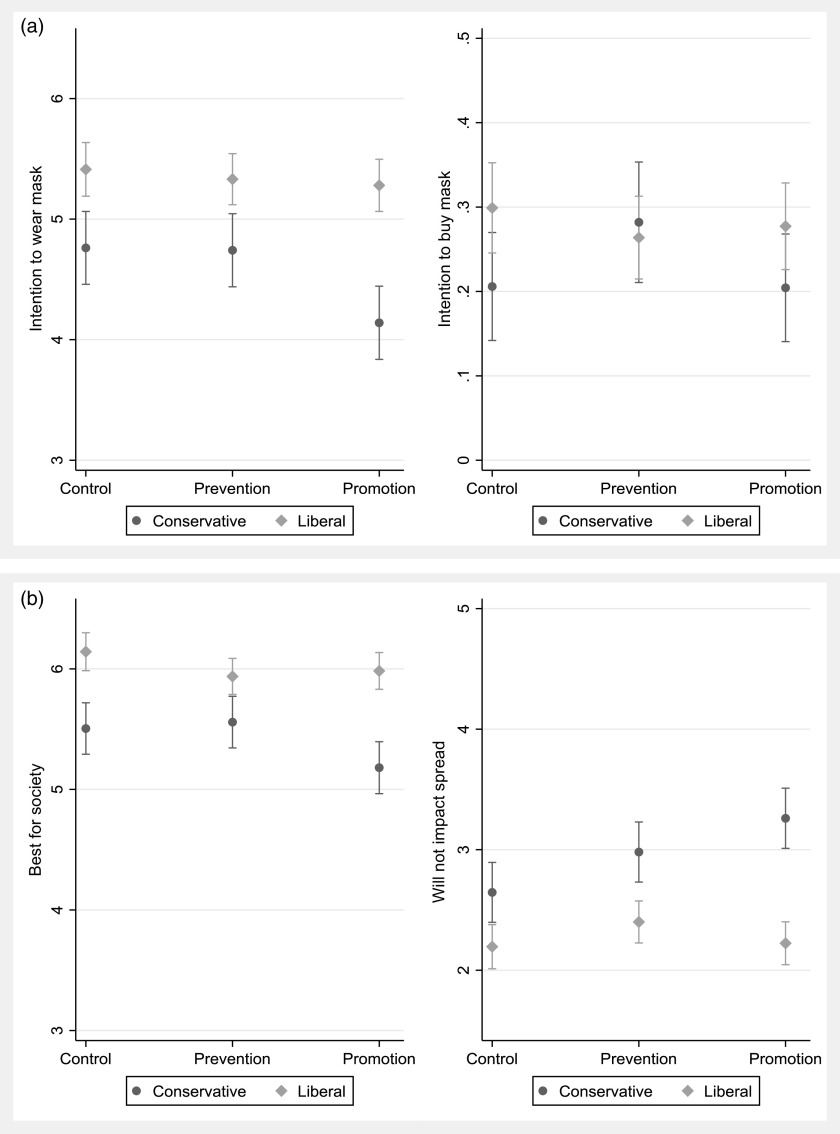
Ideology and Mask Wearing (Binary Measure). (a) Behavioral measures. (b) Attitudinal measures.

**Figure 2:**
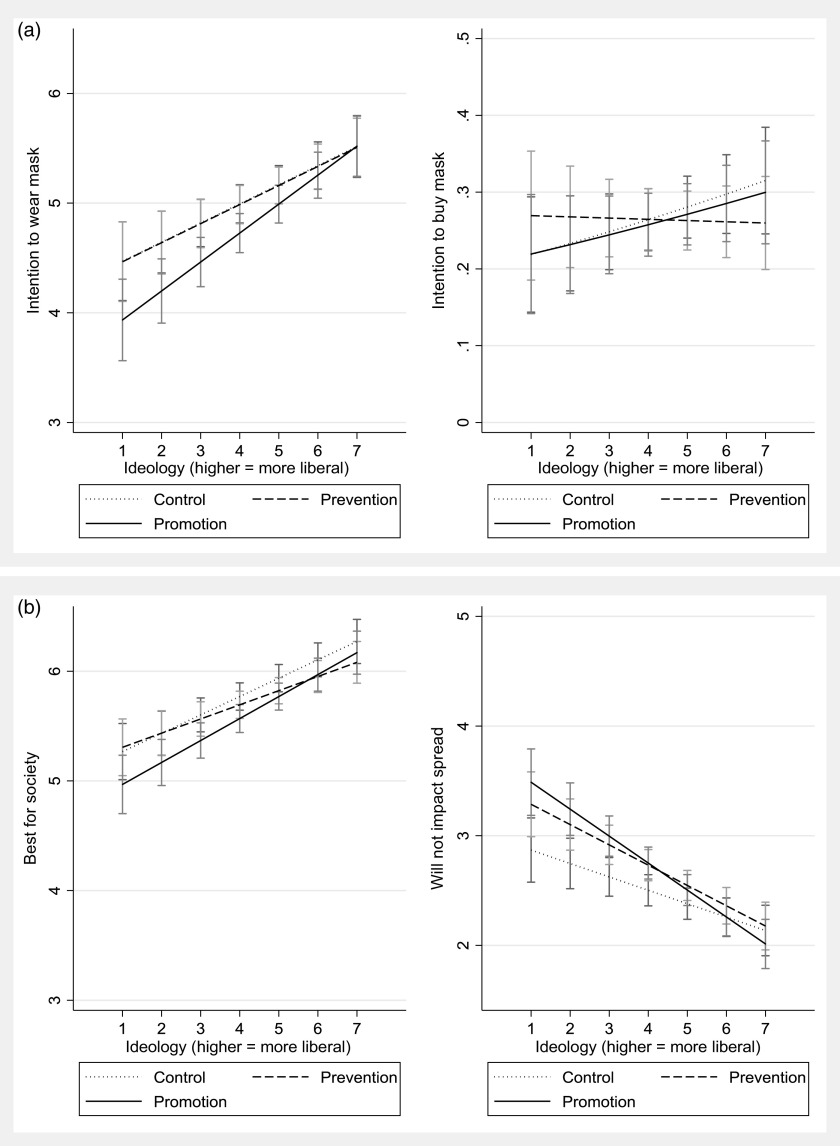
Ideology and Mask Wearing (Continuous Measure). (a) Behavioral measures. (b) Attitudinal measures.

**Figure 3:**
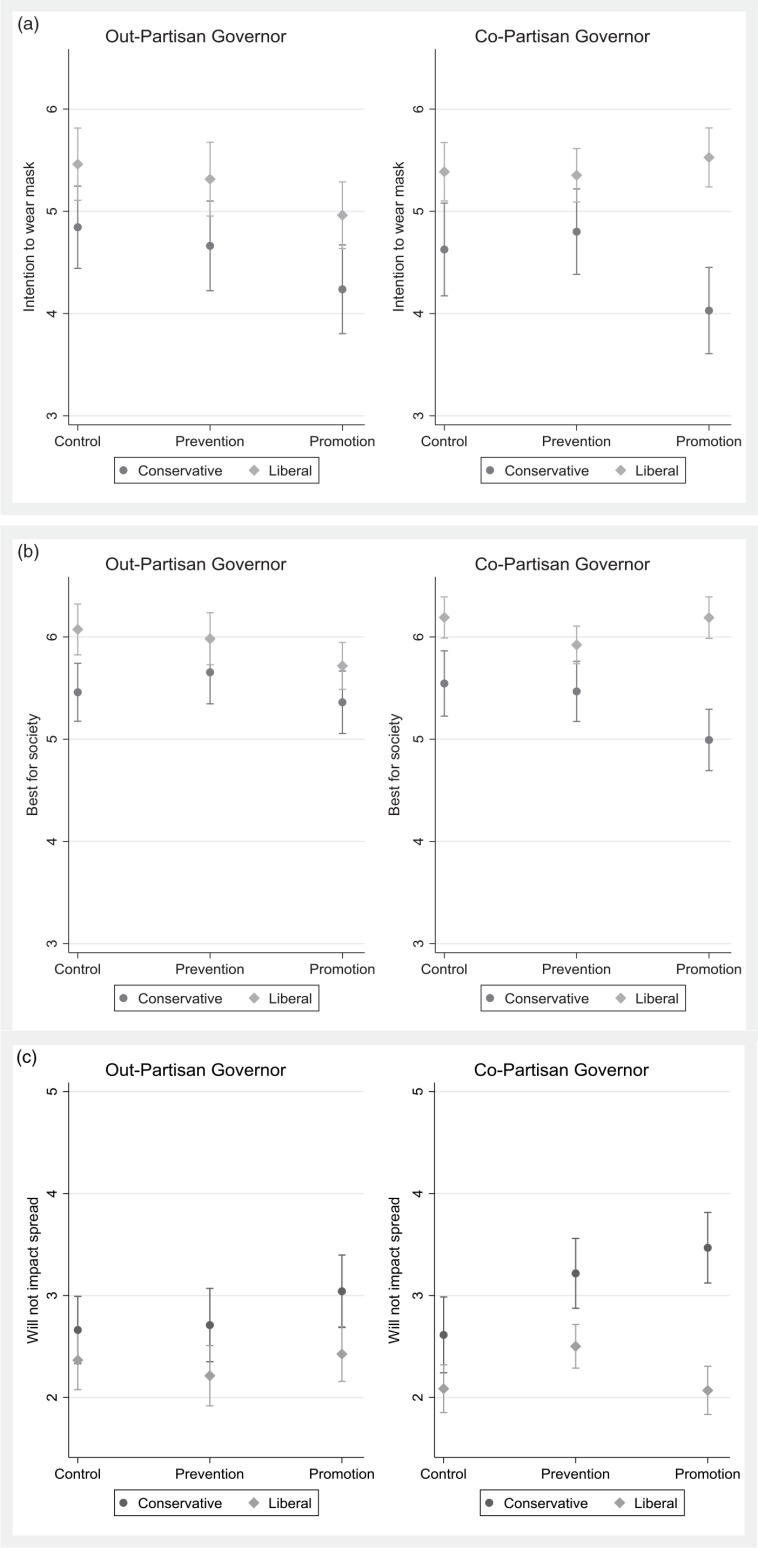
Ideology and Mask Wearing (by Governor’s Partisanship). (a) Wear mask. (b) Best for society. (c) Will not impact spread.

The results of the first set of analyses, based on a binary ideological indicator, are presented graphically in Figure 1.^5^ The results for a moderation by the seven-point ideological measure are available in Figure 2, while the results for moderation by both ideology and gubernatorial co-partisanship are presented in Figure 3. All regression models are available in the Appendix (Supplementary Material).

As shown in Figure 1, interesting, though primarily null, results emerge. There are no differences between treatments for anyone on the intention to buy mask measure – individuals were equally likely, across treatment groups and ideologies, to request information on where to purchase a mask. The behavioral intention variable, however, shows that the promotion message has an effect on desire to wear a mask. However, contrary to expectations, this does not *increase* the willingness of liberals but *decreases* the willingness of conservatives to wear a mask, relative to both the control and prevention message. Similar patterns emerge for the attitudinal measures – liberals are not affected significantly by the treatments, but conservatives are less likely to see the benefit of masks, and more likely to believe they will not impact the spread, when exposed to the promotion message, compared to the control and the prevention message.

These results are substantively similar, though a bit more difficult to digest and less statistically precise. The gap between treatments still tends to dampen for liberals and is larger for conservatives but does not reach conventional levels of statistical significance. It appears, however, that the effect of messages is more conditional upon direction of ideology, rather than strength, as no major differences in treatment effects emerge for strong and slight conservatives.

These results show little difference based on shared partisanship with one’s governor for conservatives – regardless of partisanship of a conservative respondent’s governor, they respond to the messages in similar fashion, and the promotion message still seems to dampen their compliance with, and favorability of, the mask wearing measure. For liberals, however, a different pattern emerges, in line with expectations from RFT. When liberals are exposed to the promotion message, their attitudes toward mask wearing change, compared to the other messages, dependent upon their governor’s partisanship. Liberals are more likely to believe that wearing masks is best for society and is less likely to believe that it will not impact the spread of coronavirus when exposed to the promotion message, but *only* when their governor shares their partisanship. These differences disappear when their governor does not share their partisanship.

## Discussion and Conclusion

These analyses find little support for how RFT can inform our messaging about mask wearing during the COVID-19 crisis. While messages related to prevention seem to have no impact on behaviors and attitudes toward wearing facial masks, regardless of individual ideology, promotion related messages do have some impact, though in unpredicted ways. RFT argues that liberals should be more receptive to promotion messages, and I find some very limited support of this prediction respond *positively* to promotion messages, under a specific condition – that they share partisanship with their state’s governor. However, the most consistent effects occur with conservatives showing *less* support for mask wearing when exposed to the promotion message – a finding not in line with predictions, which predict that conservatives should not be affected differently by this message than the control message.

Why are prevention messages no more (or less) effective than a purely information message? While this study is unable to examine the exact mechanism, perhaps prevention messages simply do not work in this context. Official and unofficial messaging about the COVID-19 crisis has heavily focused on prevention – “flattening the curve” is about stopping and slowing infections, an inherently prevention focused message. Perhaps the focus on prevention messages in general has rendered additional information about prevention moot, or perhaps individuals think of the mask wearing recommendation as prevention message by default.

In general, this provides little support for targeting prevention and promotion messages based on ideology, as RFT would suggest, related to mask wearing in response to the COVID-19 crisis. It seems that, generally, control messaging (without a promotion or prevention cue) and prevention messaging work equally well at promoting pro-mask attitudes and behaviors. While a difference emerges related to promotion messaging, it is unexpected, and not in line with RFT predictions, serving to dampen support from conservatives. Further work should examine this unexpected finding, to determine whether it is specifically related to the COVID-19 crisis, or if it broadly extends to how conservatives receive promotion focused messages.

This leaves policy recommendations a bit muddled – to the extent there is an impact, it is that promotion messages make conservatives *less* complaint. This suggests that a purely informational message, without an additional prod, may be the most effective strategy – since the evidence here provides little support for RFT theories of ideological differences in response to messaging about mask wearing during the COVID-19 crisis, it is likely easiest to provide a straightforward message.

## Footnotes

1
https://www.cnn.com/2020/03/23/us/coronavirus-which-states-stay-at-home-order-trnd/index.html

2
https://www.goodhousekeeping.com/health/wellness/a32038711/should-i-wear-face-masks-to-grocery-stores-coronavirus/

3 While MTurk may not provide the best sample, trading off costs with other samples makes it appropriate, especially for experimental research. Using recommendations on best practices for avoiding low-quality responses (see Kennedy et al., 2018) can significantly increase the quality of data obtained from MTurk, which comes at a fraction of the price of other sampling methods.

4 These include a brief English language test question, where participants were asked to solve a simple math word problem. Additionally, I ask participants to identify a picture of an eggplant, which has different primary words to describe it in the USA and India, to further screen. Additional requirements on MTurk use their screening functions, where individuals must be listed as located in the USA, over 18 years old, have over 100 HITs approved and have a 95% or greater approval rating on all tasks (see Kennedy et al., 2018 for these recommendations). Lastly, I use advice from Kennedy et al. (2018) on IP address screening in Qualtrics to ensure individuals are not coming from outside of the USA.

5 All figures error bars indicating 95% one-tailed confidence intervals around the estimate.

## Supplementary material

For supplementary material accompanying this paper visit http://dx.doi.org/10.1017/XPS.2020.15. click here to view supplementary material
