# Supplementary material for: Messaging Mask Wearing During the COVID-19 Crisis: Ideological Differences
Source: Journal of Experimental Political Science. 2020 May 27:1–11. doi: 10.1017/XPS.2020.15 (PMC7322225; doi:10.1017/XPS.2020.15)
Supplement: Supplementary file 1 [file S2052263020000159sup001.docx]

**Supplemental Appendix for Messaging mask wearing during the COVID-19 crisis: Ideological differences**

Stephen M. Utych

Boise State University

**Experimental Texts**

Control:

*The Centers for Disease Control (CDC) has recently recommended that all Americans wear a facial mask when leaving their homes, in order to prevent the spread of the coronavirus.*

Prevention:

*The Centers for Disease Control (CDC) has recently recommended that all Americans wear a facial mask when leaving their homes, in order to prevent the spread of the coronavirus.*

*Following this recommendation will help us prevent Americans from dying, and prevent our economy from entering a long-term recession.*

Promotion:

*The Centers for Disease Control (CDC) has recently recommended that all Americans wear a facial mask when leaving their homes, in order to prevent the spread of the coronavirus.*

*Following this recommendation will help us to keep Americans healthy, and allow our economy to return to normal more quickly.*

**Ideology measure.**

We hear a lot of talk these days about liberals and conservatives.  Here is a seven-point scale on which the political views that people might hold are arranged from extremely liberal to extremely conservative.  Where would you place yourself on this scale, or haven't you thought much about this?

- Very liberal (1)
- Liberal (2)
- Somewhat liberal (3)
- Moderate / Middle of the road (4)
- Somewhat conservative (5)
- Conservative (6)
- Very conservative (7)
- I haven't thought much about this (8)

**Covariate question texts**

[age] What is your age in years?

textbox numerical response

if age<18: **Terminate survey.** *Sorry, if you are under 18, you cannot participate in the survey.*

[gender] What is your gender?

Female

Male

Other __________

[race] What racial or ethnic group best describes you?

Asian

African-American / Black

Hispanic / Latino

Native American

White

Other [specify]

[education] What is the highest level of education that you have earned?

[DROP-DOWN]

8th grade

Attended high school

High school degree or equivalent

Associate’s degree

Some college

Bachelor’s degree

Advanced degree

[pID]: Generally speaking, do you usually think of yourself as a Republican, a Democrat, an Independent, or what?

Republican

Democrat

Independent

Other

[rep1]: [If pID = Republican] Would you call yourself a strong Republican or a not very strong Republican?

Strong Republican

Not a very strong Republican

[dem1]: [If pID = Democrat] Would you call yourself a strong Democrat or a not very strong Democrat?

Strong Democrat

Not a very strong Democrat

[ind1]: [If pID = Independent or Other] Do you think of yourself as closer to the Republican or Democratic party?

Republican party

Democratic party

Neither party

**Dependent Variables**

Please rate the extent to which you agree or disagree with the following statements…

[wearmask] I will wear a mask every time I leave my home.

[bestsoc] It is best for society if everyone wears a mask when they leave their home

[notprevent] I don’t think wearing a mask will impact the spread of the coronavirus

Strongly agree

Agree

Somewhat agree

Neither agree nor disagree

Somewhat disagree

Disagree

Strongly agree

Would you like to receive more information about purchasing face masks at the end of this survey?

Yes

No

**Table A1.** *Treatment Effects Conditional on Binary Ideology*

|  | Wear Mask | Purchase Mask | Best for Society | Won’t Impact Spread |
| --- | --- | --- | --- | --- |
|  |  |  |  |  |
| Prevention | -0.020 (0.259) | 0.425 (0.326) | 0.053 (0.182) | 0.335 (0.212) |
| Promotion | -0.621** (0.259) | -0.010 (0.343) | -0.325* (0.183) | 0.615** (0.213) |
| Liberal | 0.651** (0.229) | 0.510* (0.291) | 0.637** (0.161) | -0.451** (0.188) |
| Prevention x Liberal | -0.061 (0.318) | -0.604 (0.394) | -0.258 (0.224) | -0.129 (0.261) |
| Promotion x Liberal | 0.489 (0.320) | -0.099 (0.409) | 0.165 (0.226) | -0.586* (0.263) |
| Age | 0.029** (0.005) | 0.014* (0.006) | 0.011** (0.004) | -0.022** (0.004) |
| Female | 0.308** (0.125) | 0.031 (0.153) | 0.164* (0.088) | -0.160 (0.103) |
| Black | 0.755** (0.282) | 1.106** (0.307) | 0.559** (0.199) | -0.375 (0.232) |
| Latino/a | 0.613* (0.278) | -0.403 (0.383) | 0.081 (0.198) | 0.013 (0.228) |
| Other race | 0.796** (0.200) | 0.076 (0.243) | 0.305* (0.141) | -0.398** (0.165) |
| Education | -0.018 (0.052) | 0.073 (0.065) | 0.015 (0.037) | 0.006 (0.043) |
| Co-Partisan Governor | 0.086 (0.126) | -0.028 (0.154) | 0.062 (0.089) | 0.029 (0.103) |
| Constant | 3.348** (0.393) | -2.352** (0.505) | 4.799** (0.278) | 3.585** (0.323) |
| *N* | 940 | 940 | 939 | 939 |
| *(pseudo) R*^2^ | 0.1020 | 0.0243 | 0.0775 | 0.0809 |

Table entries are OLS (Columns 1, 3,4 ) or logit (Column 2) coefficients with standard errors in parentheses

* p<0.05, ** p<0.01, one-tailed

**Table A2.** *Treatment Effects Conditional on Continuous Ideology*

|  | Wear Mask | Purchase Mask | Best for Society | Won’t Impact Spread |
| --- | --- | --- | --- | --- |
|  |  |  |  |  |
| Prevention | -0.005 (0.377) | 0.377 (0.466) | 0.077 (0.269) | 0.481 (0.308) |
| Promotion | -0.625 (0.382) | 0.017 (0.479) | -0.332 (0.273) | 0.743** (0.311) |
| Liberal | 0.174** (0.055) | 0.085 (0.068) | 0.167** (0.039) | -0.122** (0.045) |
| Prevention x Liberal | -0.001 (0.076) | -0.094 (0.093) | -0.038 (0.054) | -0.063 (0.062) |
| Promotion x Liberal | 0.090 (0.077) | -0.013 (0.095) | 0.033 (0.055) | -0.124* (0.063) |
| Age | 0.029** (0.005) | 0.015** (0.006) | 0.013** (0.003) | -0.023** (0.004) |
| Female | 0.263* (0.118) | 0.056 (0.143) | 0.081 (0.085) | -0.075 (0.097) |
| Black | 0.911** (0.269) | 1.203** (0.290) | 0.672** (0.192) | -0.471* (0.219) |
| Latino/a | 0.696** (0.274) | -0.300 (0.366) | 0.161 (0.197) | -0.078 (0.224) |
| Other race | 0.853** (0.185) | 0.321 (0.215) | 0.422** (0.132) | -0.526** (0.151) |
| Education | -0.009 (0.049) | 0.092 (0.061) | 0.021 (0.035) | 0.001 (0.040) |
| Co-Partisan Governor | 0.058 (0.120) | -0.049 (0.145) | 0.048 (0.085) | 0.013 (0.098) |
| Constant | 2.863** (0.420) | -2.561** (0.529) | 4.333** (0.300) | 3.993** (0.342) |
| *N* | 1054 | 1053 | 1053 | 1053 |
| *(pseudo) R*^2^ | 0.0997 | 0.0253 | 0.0824 | 0.0844 |

Table entries are OLS (Columns 1, 3,4 ) or logit (Column 2) coefficients with standard errors in parentheses

* p<0.05, ** p<0.01, one-tailed

**Table A3.** *Treatment Effects Conditional on Ideology and Co-Partisanship of Governor*

|  | Wear Mask | Purchase Mask | Best for Society | Won’t Impact Spread |
| --- | --- | --- | --- | --- |
|  |  |  |  |  |
| Prevention | -0.182 (0.361) | 0.372 (0.446) | 0.196 (0.254) | 0.048 (0.296) |
| Promotion | -0.607* (0.359) | 0.029 (0.461) | -0.098 (0.253) | 0.379 (0.294) |
| Liberal | 0.617* (0.326) | 0.600 (0.400) | 0.615** (0.229) | -0.296 (0.267) |
| Co-Partisan Governor | -0.218 (0.368) | -0.251 (0.492) | 0.086 (0.259) | -0.048 (0.302) |
| Prevention x Liberal | 0.036 (0.474) | -0.760 (0.575) | -0.286 (0.334) | -0.201 (0.389) |
| Promotion x Liberal | 0.108 (0.461) | -0.643 (0.579) | -0.259 (0.325) | -0.319 (0.378) |
| Prevention x Co-Partisan Gov | 0.357 (0.519) | 0.137 (0.658) | -0.273 (0.365) | 0.555 (0.425) |
| Promotion x Co-Partisan Gov | 0.010 (0.521) | -0.061 (0.693) | -0.455 (0.368) | 0.475 (0.427) |
| Control x Lib x CPG | 0.143 (0.460) | -0.087 (0.587) | 0.031 (0.324) | -0.232 (0.376) |
| Prevent x Lib x CPG | -0.101 (0.458) | 0.128 (0.552) | 0.127 (0.323) | -0.218 (0.375) |
| Promote x Lib x CPG | 0.773* (0.451) | 0.838 (0.587) | 0.841** (0.318) | -0.783* (0.370) |
| Age | 0.030** (0.005) | 0.014** (0.006) | 0.011** (0.004) | -0.021** (0.004) |
| Female | 0.312** (0.125) | 0.034 (0.154) | 0.168* (0.088) | -0.169* (0.103) |
| Black | 0.771** (0.284) | 1.114** (0.310) | 0.586** (0.200) | -0.423* (0.232) |
| Latino/a | 0.612* (0.278) | -0.408 (0.384) | 0.069 (0.198) | 0.022 (0.228) |
| Other race | 0.775** (0.200) | 0.044 (0.245) | 0.286* (0.141) | -0.384** (0.165) |
| Education | -0.020 (0.053) | 0.068 (0.066) | 0.014 (0.037) | 0.007 (0.043) |
| Constant | 3.475** (0.419) | -2.256** (0.537) | 4.794** (0.295) | 3.606** (0.343) |
| *N* | 940 | 940 | 939 | 939 |
| *(pseudo) R*^2^ | 0.1069 | 0.0284 | 0.0866 | 0.0915 |

Table entries are OLS (Columns 1, 3,4 ) or logit (Column 2) coefficients with standard errors in parentheses

* p<0.05, ** p<0.01, one-tailed
